# Gi- and Gq-coupled ADP (P2Y) receptors act in opposition to modulate nociceptive signaling and inflammatory pain behavior

**DOI:** 10.1186/1744-8069-6-21

**Published:** 2010-04-15

**Authors:** Sacha A Malin, Derek C Molliver

**Affiliations:** 1 Dept Medicine; Dept Neurobiology, University of Pittsburgh, Pittsburgh, PA, USA

## Abstract

**Background:**

Investigations of nucleotide signaling in nociception to date have focused on actions of adenosine triphosphate (ATP). Both ATP-gated ion channels (P2X receptors) and G protein-coupled (P2Y) receptors contribute to nociceptive signaling in peripheral sensory neurons. In addition, several studies have implicated the Gq-coupled adenosine diphosphate (ADP) receptor P2Y1 in sensory transduction. In this study, we examined the expression and function of P2Y1 and the Gi-coupled receptors P2Y12, P2Y13 and P2Y14 in sensory neurons to determine their contribution to nociception.

**Results:**

We detected mRNA and protein for ADP receptors P2Y12 and P2Y13 in mouse dorsal root ganglia (DRG). P2Y14, a homologous Gi-coupled nucleotide receptor, is also expressed in DRG. Immunohistochemical analysis of receptor distribution indicated that these receptors are widely expressed in nociceptive neurons. Using ratiometric calcium imaging, we found that ADP evokes increases in intracellular calcium in isolated DRG neurons and also produces a pertussis toxin-sensitive inhibition of depolarization-evoked calcium transients. The inhibitory effect of ADP was unaltered in the presence of the selective P2Y1 antagonist MRS2179 and in neurons isolated from P2Y1 knockout mice, whereas ADP-evoked calcium transients were greatly reduced. Analysis of behavioral responses to noxious heat before and after inflammatory injury (injection of complete Freund's adjuvant into the hindpaw) revealed that P2Y1 is required for the full expression of inflammatory hyperalgesia, whereas local injection of agonists for Gi-coupled P2Y receptors reduced hyperalgesia.

**Conclusions:**

We report that Gi-coupled P2Y receptors are widely expressed in peripheral sensory neurons. Agonists for these receptors inhibit nociceptive signaling in isolated neurons and reduce behavioral hyperalgesia *in vivo*. Anti-nociceptive actions of these receptors appear to be antagonized by the Gq-coupled ADP receptor, P2Y1, which is required for the full expression of inflammatory hyperalgesia. We propose that nociceptor sensitivity is modulated by the integration of nucleotide signaling through Gq- and Gi-coupled P2Y receptors, and this balance is altered in response to inflammatory injury. Taken together, our data suggest that Gi-coupled P2Y receptors are broadly expressed in nociceptors, inhibit nociceptive signaling *in vivo*, and represent potential targets for the development of novel analgesic drugs.

## Background

Adenosine triphosphate (ATP) has been studied for more than 30 years as a potential nociceptive messenger [1]. Extensive investigation into the role of purinergic signaling in nociception was stimulated by the identification of ATP-gated ion channels (the P2X receptors) in primary afferent nociceptors. More recently, several groups have implicated members of the P2Y family of G protein-coupled nucleotide receptors in sensory transduction [2,3]. In contrast to the ATP-selective (purinergic) P2X receptors, the 8 known P2Y receptors respond to a variety of purine and pyrimidine nucleotides (Table 1). In sensory neurons, activation of the Gq-coupled receptor P2Y2 by ATP and/or UTP causes the release of intracellular calcium stores, action potential firing, the release of neuropeptides and activation of the transcription factor CREB ([4-6]). The ADP receptor P2Y1 is also expressed in sensory neurons and has been implicated in sensory transduction [7,8]. Evidence for the expression and function of other P2Y receptors in sensory neurons is limited [2,9].

**Table 1:** P2Y receptors and nucleotide agonists.

Receptor	Signaling	Preferred Agonist	ΔC_T _(DRG)
P2Y1*	Gq/11	ADP	6.22 ± 0.11

P2Y2*	Gq/11	ATP = UTP	6.17 ± 0.08

P2Y4*^‡^	Gq/11	ATP = UTP	13.63 ± 0.31

P2Y6*	Gq/11	UDP	10.92 ± 0.14

P2Y12	Gi/o	ADP	7.55 ± 0.26

P2Y13**	Gi/o	ADP = IDP	9.78 ± 0.59

P2Y14	Gi/o	Glycosylated UDP	7.91 ± 0.59

Relative expression levels of mRNA for P2Y family members in DRG are expressed as the normalized cycle threshold (ΔC_T_): the P2Y C_T _value minus the C_T _for the reference standard, GAPDH. *Previously published data [28]. ^‡^Rodent P2Y4 receptor shows equivalent responsiveness to ATP and UTP; the human receptor is selective for UTP. **Mouse but not human P2Y13 shows high affinity for IDP as well as ADP. P2Y11 is not included here because it is not expressed in rodents. (See [10,37-39]).

The recently-identified P2Y receptors P2Y12, P2Y13 and P2Y14 represent a subfamily of nucleotide GPCRs with only limited homology to the Gq-coupled P2Y receptors and distinct signal transduction mechanisms via Gi/o [10]. For convenience, these three receptors will be collectively referred to here as P2Y_Gi _receptors. P2Y12 and P2Y13 are preferentially activated by ADP, whereas P2Y14 is selective for glycosylated UDP. Although these compounds are metabolites of ATP and UTP and thus present in the sensory neuron milieu, the extent to which these receptors and their ligands contribute to sensory signal transduction remains under active investigation. Activation of Gi-coupled receptors in sensory neurons is often associated with inhibition of N-type Ca^++ ^channels and attenuation of neurotransmitter release, which is the principle mechanism for the inhibition of peripheral nociceptive signaling by mu opioid receptor agonists [11,12]. This phenomenon has been demonstrated using fura-2 Ca^++ ^imaging to visualize the inhibition of depolarization-evoked Ca^++ ^influx by opioid agonists in dissociated DRG neurons [13,14]. Here, we demonstrate that P2Y12, P2Y13 and P2Y14 are all expressed in sensory neurons of the dorsal root ganglia (DRG) with neurochemical characteristics of nociceptors. Ligands for each of these receptors inhibit depolarization-evoked influx of extracellular Ca^++^. Expression of all three Gi-coupled nucleotide receptors is regulated in response to inflammation, indicating that changes in P2Y_Gi _expression contribute to the neuronal response to inflammatory injury. Results described here indicate that ADP acts at both the Gq-coupled P2Y1 and the Gi-coupled P2Y12 and P2Y13 receptors in sensory neurons, and suggest that the integration of these antagonistic pathways is an important mechanism for the modulation of nociceptor sensitivity.

## Results

**Figure 1:**
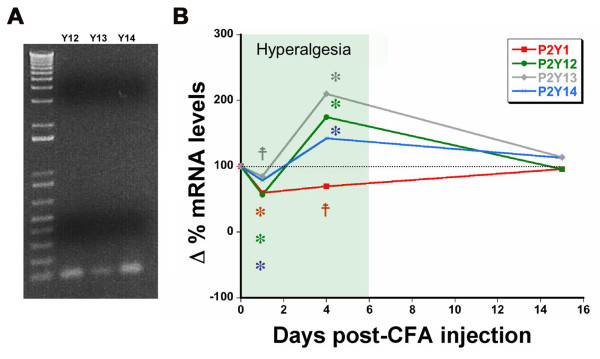
**P2Y_Gi _receptor mRNA levels are regulated in response to inflammatory injury**. **A**) Amplification of DRG cDNA by conventional RT-PCR using the P2Y_Gi _real-time PCR primers produced a single band for each receptor of the expected size (100-150 bp). **B**) Real-time PCR was used to analyze mRNA levels for each of the Gi-coupled P2Y receptors and the Gq-coupled P2Y1 at 1, 4 and 15 days after induction of inflammatory hyperalgesia by injection of complete Freund's adjuvant into the hindpaw. The shaded box indicates the period of significant heat hyperalgesia determined using the Hargreaves test. Data are normalized against baseline values. *p < 0.01, ^‡^p < 0.05, For PCR, n = 5 mice/time point. For behavior, n = 10 mice/time point.

Expression of mRNA for all three of the Gi-coupled P2Y receptors was identified in mouse lumbar DRG by real-time PCR, with relative levels of expression of P2Y12 ~P2Y14 > P2Y13 (Table 1). To determine whether expression is altered in response to inflammatory injury, 20 μl complete Freund's adjuvant (CFA) was injected into the plantar surface of the hindpaw, and real-time PCR was used to quantify changes in mRNA abundance compared to uninjected baseline levels (Figure 1). The three P2Y_Gi _receptors were coordinately regulated: expression was initially reduced one day after CFA injection, but significantly upregulated at day 4. Expression of P2Y_Gi _receptor mRNA returned to baseline by day 15, by which time behavioral response thresholds to noxious heat had also returned to baseline levels.

**Figure 2:**
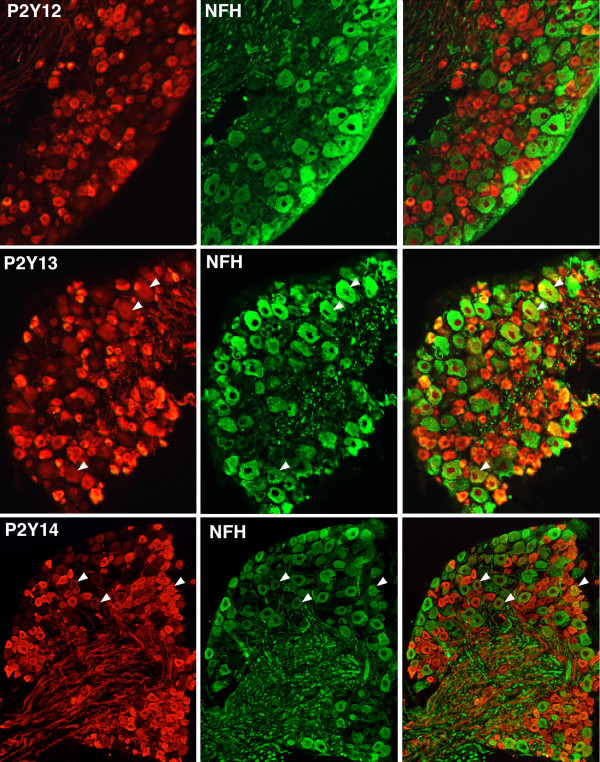
**P2Y_Gi _receptor immunoreactivity is localized in sensory neurons of the DRG**. Staining for P2Y12 (**A**), P2Y13 (**B**) and P2Y14 (**C**) in cryostat sections of DRG neurons double-labeled for the high-molecular weight neurofilament protein, NFH, used as a marker of neurons with myelinated axons. Antibodies for all three receptors intensely stain small-diameter, NFH-negative neurons. However, P2Y12 expression is largely restricted to NFH-negative neurons, whereas P2Y13 and P2Y14 are expressed in some NFH-positive sensory neurons (arrowheads). n = 3 mice, > 400 cells/antibody.

Immunohistochemistry for all three P2Y_Gi _receptors revealed intense staining in the great majority of small-diameter neurons, consistent with widespread expression in nociceptors (Figure 2). Although P2Y12 immunoreactivity was restricted to small neurons, both P2Y13 and P2Y14 were detected in some larger neurons. Staining was less intense in large neurons. Consistent with this observation, only 4.2 ± 2.0% of P2Y12-positive neurons were positive for neurofilament, a marker for neurons with myelinated axons [15], while 17.5 ± 4.3% of P2Y13-positive and 29.5 ± 5.1% of P2Y14-positive neurons displayed immunoreactivity for neurofilament (Figure 2).

As is evident in Table 1, P2 receptors have a high degree of agonist promiscuity, which has hindered analysis of receptor-specific effects. To determine whether P2Y_Gi _receptors are capable of modulating depolarization-evoked Ca^++ ^transients, we used ratiometric Ca^++ ^imaging to measure responses to a combination of agonists and antagonists in neurons isolated from wildtype and P2Y1-/- mice. Both P2Y12 and P2Y13 are activated by ADP [16], but, P2Y13 is the only P2Y receptor with high affinity for IDP. P2Y14 is unique in that it is highly selective for glycosylated UDP [10]. Therefore, we compared the actions of ADP, IDP and UDP-glucose (UDPG) on isolated mouse DRG neurons. Because ADP is also an agonist for the Gq-coupled P2Y1 receptor, we characterized agonist effects in neurons from both wildtype and P2Y1-/- mice.

Application of ADP (100 μM) to isolated wildtype DRG neurons evoked a transient increase in intracellular Ca^++ ^in roughly half of all neurons, consistent with the activation of Gq-coupled signaling (Table 2). This proportion dropped to less than 10% of neurons in the presence of the selective P2Y1 antagonist MRS2179, and of neurons isolated from P2Y1-/- mice. These data indicate that most ADP responses *in vitro *were mediated by P2Y1. When the concentration of ADP was reduced to 10 μM, the percentage of responsive neurons dropped to 35%; the magnitude of responses also decreased, indicating submaximal activation of P2Y1 signaling. These results support the functional expression of P2Y1 in approximately 40% of DRG neurons *in vitro*.

**Table 2:** ADP evokes Ca^++ ^transients through P2Y1.

	10 μM ADP	100 μM ADP	MRS2179 100 μM ADP
**wildtype**			
% responders	35 ± 5%	52 ± 6%	8 ± 3%
n (cells)	37	197	40
**wildtype**			
response size (ΔF)	0.09 ± 0.01	0.53 ± 0.05	0.18 ± 0.02
**wildtype**			
response area (ΔF_area_)	1 ± 2	11 ± 2	6 ± 1

**P2Y1-/-**			
% responders	ND	9 ± 4%	10 ± 1%
n (cells)		100	55
**P2Y1-/-**			
response size (ΔF)	ND	0.90 ± 0.28	1.31 ± 0.32
**P2Y1-/-**			
response area (ΔF_area_)	ND	15 ± 3	18 ± 4

Dramatically fewer sensory P2Y1-/- neurons produce ADP-evoked calcium transients compared to wildtype neurons. Remaining responses in P2Y1-/- neurons are similar in size to those seen in wildtype. Similar effects were obtained using MRS2179, a P2Y1-specific antagonist. Frequency and response magnitude values are mean ± SEM.

**Figure 3:**
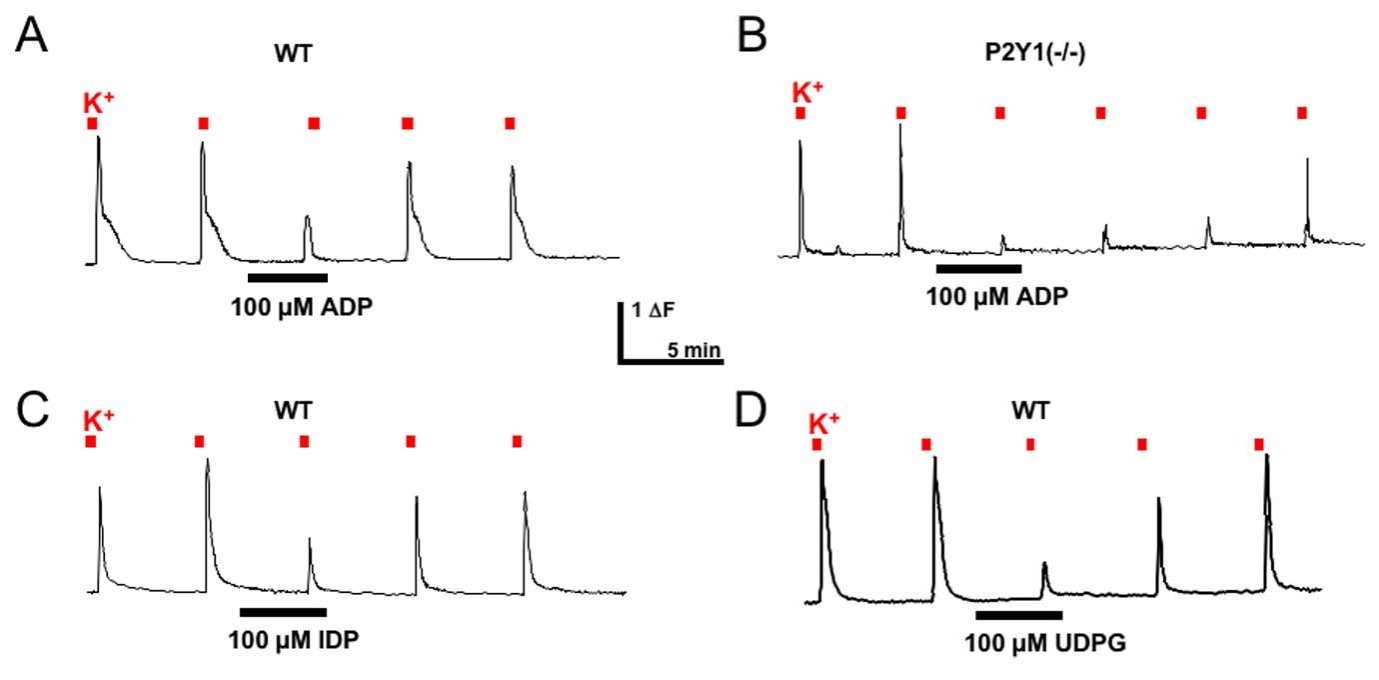
**P2Y_Gi _agonists inhibit depolarization-evoked increases in intracellular Ca^++^**. Fura-2 Ca^++ ^imaging was used to measure the effect of P2Y receptor agonists on Ca^++ ^transients evoked by administration of 50 mM K^+^. Application of ADP (100 μM) for 3 minutes reduced subsequent depolarization-evoked transients in wildtype (**A**) and P2Y1-/- (**B**) mice. The P2Y13 agonist IDP (**C**) and the P2Y14 agonist UDPG (**D**) also inhibited depolarization-evoked transients.

To examine the actions of the P2Y_Gi _receptors, we first tested the ability of ADP to inhibit Ca^++ ^transients evoked by a depolarizing stimulus of 50 mM K^+^. ADP (100 μM) inhibited both the magnitude and the duration of depolarization-evoked transients in the majority of neurons *in vitro *(Figure 3A). ADP was equally effective in neurons from P2Y1-/- mice, ruling out an essential role for P2Y1 (Figure 3B). The inhibitory effect of ADP was lost when cells were pretreated overnight with 250 nM pertussis toxin, indicating that inhibition was mediated through a Gi-coupled pathway (n = 3 mice, 32 cells, data not shown). IDP and UDPG also inhibited depolarization-evoked Ca^++ ^transients (Figure 3C-D; Table 3).

**Table 3:** Extent of inhibition by P2Y_Gi _receptor agonists.

Agonist (genotype)	ADP (wt)	IDP (wt)	UDPG (wt)	ADP (P2Y1-/-)
**All DRG**				
Inhibition frequency	65 ± 7%	73 ± 4%	66 ± 10%	75 ± 7%
Inhibition magnitude	48 ± 5%	33 ± 4%	38 ± 4%	44 ± 4%
n(cells)	185	48	120	112

**TRPV1+ DRG**				
Inhibition frequency	59 ± 9%	88 ± 13%	82 ± 8%	61 ± 14%
Inhibition magnitude	34 ± 22%	31 ± 6%	56 ± 10%	47 ± 9%
n(cells)	112	30	70	72

P2Y_Gi _receptor agonists inhibit depolarization-evoked Ca^++ ^transients in sensory neurons at 5 minutes after application. Table shows the percentage of sensory neurons in wild type and P2Y1-/- mice inhibited by each agonist and the magnitude of inhibition (change in peak response); values are given as mean ± SEM. Data are given for all sensory neurons, as well as for capsaicin-responsive cells (TRPV1+).

**Figure 4:**
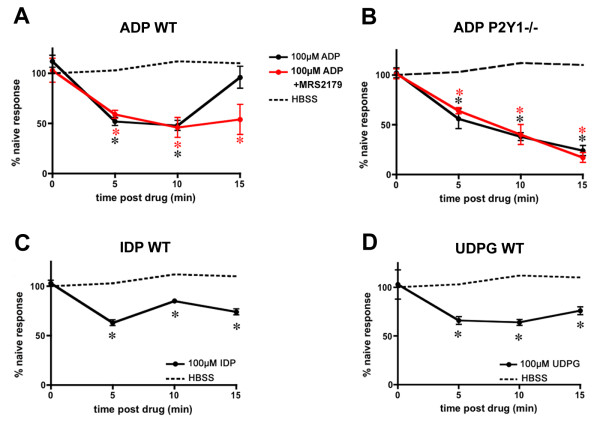
**The inhibitory effect of ADP is enhanced in the absence of P2Y1 signaling**. The magnitudes of depolarization-evoked Ca^++ ^transients in sensory neurons were measured before, and 5, 10 and 15 minutes after agonist application. Ca^++ ^transients were not affected by application of buffer alone (dashed lines), but were significantly reduced after application of ADP (**A-B**), IDP (**C**), or UDPG (**D**; solid lines). Inhibition was prolonged by application of the selective P2Y1 antagonist MRS2179 (**A**) and in neurons from P2Y1-/- mice (**B**). There was no effect of MRS2179 in P2Y1-/- neurons (**B**). Values are mean ± SEM; n = 10 mice/timepoint each treatment. *p < 0.02 versus control for each treatment.

Neurons inhibited by nucleotides were tested for responsiveness to 1 μM capsaicin to test for expression of the capsaicin receptor TRPV1 as a marker for a subset of nociceptors [17]. A large proportion of capsaicin-responsive neurons was inhibited by nucleotides, consistent with our hypothesis that P2Y_Gi _receptors have inhibitory actions in nociceptive sensory neurons (Table 3). UDPG inhibited the largest proportion of capsaicin-responsive neurons, followed by ADP and IDP. Application of ADP, IDP or UDPG resulted in long-lasting inhibition in many cells. Responses at 5, 10 and 15 minutes post-agonist application were compared to peak responses to depolarizing stimuli obtained prior to P2Y_Gi _receptor activation; these data are shown in Figure 4. This reduction in response magnitude does not reflect run-down, as cells not treated with nucleotides showed no diminution of depolarizing responses. These data suggest that P2Y_Gi _activation may have analgesic effects *in vivo*.

Surprisingly, the inhibitory effects of nucleotides on sensory neuron function were enhanced by the blockade of P2Y1. Application of MRS2179 increased both the magnitude and duration of ADP-evoked inhibition of depolarizing responses (Figure 4). Similar results were obtained from P2Y1-/- neurons (Figure 3, 4). This finding suggests that P2Y1 signaling antagonizes the action of P2Y_Gi _receptors.

**Figure 5:**
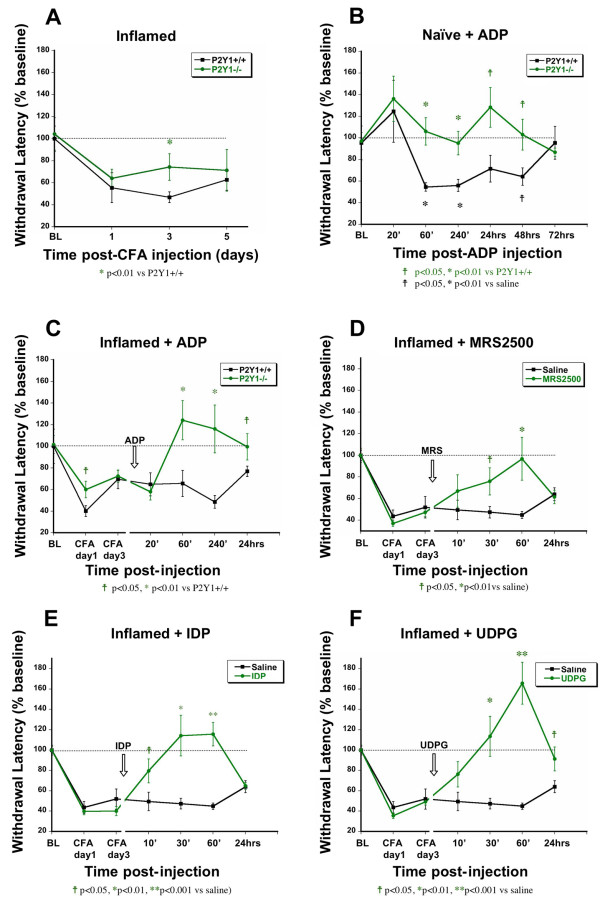
**ADP has opposing effects on behavioral nociceptive thresholds in the presence and absence of P2Y1**. ADP has opposite effects on withdrawal latencies to noxious heat stimuli (Hargreaves test) in the presence and absence of P2Y1. **A) **P2Y1-/- mice show a deficit in thermal hyperalgesia at day 3 following CFA injection, compared to wildtype mice. **B) **Injection of ADP into the hindpaw of naïve mice caused thermal hyperalgesia in wildtype mice, but hypoalgesia in P2Y1-/- mice. **C) **Mice were injected with ADP in the hindpaw three days after CFA injection and thermal response thresholds were measured. ADP injection had no effect on thermal thresholds in inflamed wildtype mice, but reversed thermal hyperalgesia in P2Y1-/- mice. **D) **Hindpaw injection of MRS2500, a P2Y1 antagonist, in inflamed wildtype mice acutely reversed thermal hyperalgesia compared to saline injection. Injection of either IDP (**E**) or UDPG (**F**) into the inflamed hindpaw reversed inflammation-evoked thermal hyperalgesia. n = 10 mice/cohort.

Our analysis of nucleotide signaling in isolated neurons suggests that P2Y_Gi _receptors have inhibitory actions in sensory neurons, whereas P2Y1 is excitatory. To determine the impact of these receptors on nociceptive signaling *in vivo*, we examined the effects of P2Y agonists on behavioral responses to noxious heat using the Hargreaves test in naïve mice and after inducing inflammation by injecting complete Freund's adjuvant (CFA) into the plantar surface of the hindpaw. Baseline paw withdrawal latencies were not different between wildtype and P2Y1-/- mice (Figure 5A), and both wildtype and mutant mice developed persistent thermal hyperalgesia after CFA injection. However, mutant mice were significantly less sensitized than wildtype at the peak of hyperalgesia on day 3, suggesting that the sensitization of nociceptors by inflammatory injury is modestly impaired in the absence of P2Y1 signaling.

We next examined the acute effect of exogenous ADP on heat sensitivity. ADP injection into the hindpaw (10 nmol/10 μl) did not cause acute nocifensive behavior, consistent with a previous report [18]. However, ADP caused a sustained decrease in withdrawal latencies to noxious heat (heat hyperalgesia) in wildtype mice that lasted up to 48 hours (Figure 5B). No hyperalgesia occurred in P2Y1-/- mice, indicating that the hyperalgesia was likely mediated by P2Y1. Strikingly, significant hypoalgesia was seen after ADP injection in P2Y1-/- mice, suggesting an antinociceptive action of other ADP receptors that was occluded in the presence of P2Y1.

To further explore the contribution of P2Y1 to inflammatory hyperalgesia, either ADP or a selective P2Y1 antagonist was injected into the inflamed paw of mice three days after CFA injection (Figure 5C-D). The P2Y1 antagonist MRS2500 (1.5 nmol/10 μl) caused a significant reduction in hyperalgesia evident at 30 and 60 minutes. ADP injection (10 nmol/10 μl) into the hindpaw 3 days after CFA did not alter hyperalgesia in inflamed wildtype mice, but caused a complete reversal of hyperalgesia in P2Y1-/- mice that lasted at least 4 hours. These results are consistent with the *in vitro *data indicating an anti-nociceptive action of ADP that was masked or antagonized in the presence of P2Y1.

Finally, P2Y_Gi _agonist IDP (P2Y13) or UDPG (P2Y14) (each at 10 nmol/10 μl) was injected into the hindpaws of wildtype mice at CFA day three. Both agonists caused temporary but complete reversal of CFA-evoked hyperalgesia lasting at least 60 minutes (Figure 5E-F). UDPG caused significant hypoalgesia at 30 and 60 minutes post-injection (mice were less sensitive to noxious heat than at baseline). This antinociceptive effect was still significant 24 hours after injection. The high efficacy of UDPG in behavioral experiments correlates well with our Ca^++ ^imaging results: UDPG showed the greatest amount of inhibition and the largest proportion of capsaicin-responsive neurons inhibited *in vitro*.

## Discussion

The identification of nucleotide receptors in nociceptive sensory neurons has spurred investigation of the mechanisms by which nucleotides contribute to nociceptive transmission. We provide evidence that P2Y1 has pro-nociceptive actions in sensory neurons and participates in inflammatory sensitization. Furthermore, we demonstrate that Gi-coupled P2Y receptors are expressed in sensory neurons, are dynamically upregulated in response to inflammation and inhibit excitatory signaling in sensory neurons, including capsaicin-responsive nociceptors.

Our results conflict with a previous report analyzing P2Y12 and P2Y14 mRNA by *in situ *hybridization in rat DRG, which concluded that radiographic silver grains observed were not localized to neuronal cell bodies [9]. Here, we used real-time PCR, immunohistochemistry and functional analysis of agonist responses using Ca^++ ^imaging and behavioral assays to document the expression of P2Y_Gi _receptors in sensory neurons from mouse DRG. Antibodies to each of the receptors revealed intense neuronal labeling. All three receptors showed particularly intense labeling in small-diameter neurons, which comprise the majority of nociceptors. This interpretation is supported by the demonstration of P2Y_Gi _agonist-evoked inhibition of Ca^++ ^transients in many capsaicin-responsive (and thus presumably nociceptive) sensory neurons. Less-intense P2Y13 and P2Y14 immunoreactivity in larger neurons suggests that these receptors may contribute to functional properties of large A-fiber nociceptors and/or some non-nociceptive neurons. Finally, ganglionic mRNA levels of all three receptors were coordinately regulated in response to hindpaw injection of CFA, indicating that modulation of Gi-coupled nucleotide receptor signaling is part of the neuronal response to inflammatory injury.

Gi-coupled receptors, including opioid receptors, inhibit sensory transmission by blocking voltage-gated Ca^++ ^channels (VDCCs) that regulate neurotransmitter release both peripherally and centrally. Inhibition of depolarization-evoked Ca^++ ^influx by opioid agonists in sensory neurons has been demonstrated previously using Ca^++ ^imaging [13,14]. This most likely occurs through a direct interaction of the G protein βγ subunit and the VDCCs (Herlitze et al., 1996; Ikeda, 1996; Zamponi and Snutch, 1998). We used a similar protocol to demonstrate the ability of nucleotide agonists for P2Y12, P2Y13 and P2Y14 to inhibit Ca^++ ^transients in DRG neurons from both wildtype and P2Y1-/- mice. Similarly, previous studies have demonstrated inhibition of VDCCs and neurotransmitter release by P2Y12 and/or P2Y13 in PC12 cells and sympathetic neurons [19-21].

Gerevich et al [22] reported that P2Y1 mediates inhibition of VDCCs in isolated DRG neurons by ADP through an action of the βγ subunits of Gq. Intriguingly, these authors also demonstrated that intrathecal administration of a non-selective agonist of P2Y1, P2Y12 and P2Y13 (ADP-β-S) inhibited spinal nociceptive transmission and nocifensive behavior in the rat tail flick test [22]. Our results in P2Y1-/- mice demonstrate that P2Y1 is not necessary for the inhibition of Ca^++ ^signaling by ADP. Indeed, the inhibitory action of ADP in isolated neurons and on pain behavior *in vivo *was enhanced in the absence of P2Y1 signaling, suggesting an action of P2Y12 and/or P2Y13. However, our results suggest that the dominant effect of ADP in wildtype mice is pro-nociceptive and that this action requires P2Y1. Application of ADP to DRG neurons *in vitro *evoked increases in intracellular Ca^++^, and subcutaneous injection of ADP in wildtype mice caused heat hyperalgesia, a finding consistent with a previous report that an ADP analog enhanced heat responses in isolated polymodal nociceptors [23]. In the absence of P2Y1, ADP was anti-nociceptive, suggesting an action of P2YGi receptors that is normally occluded by P2Y1. Such interactions between Gq and Gi signaling do not appear to be unique to P2Y receptors, as a previous study found that genetic deletion of the Gq effector phospholipase C beta 3 resulted in enhanced Gi signaling through the mu opioid receptor [24].

The expression of ADP receptors in sensory neurons, in addition to ATP-gated P2Y receptors and P2X channels, begs the question of how these diverse extracellular signals are integrated in the neuron. While much work remains to be done on this issue, one key factor is the regulation of local nucleotide concentration by extracellular nucleotidases. Most nucleotidases rapidly degrade micromolar ATP but degrade ADP more slowly, resulting in the rapid termination of ATP signaling and a more persistent ADP signal; relative kinetics depend on which family members are expressed [25]. Tonic or spontaneous release of ATP might thus result in little or no ATP signaling, but tonic ADP signaling (for example, see [26]). Acute release of ATP caused, for instance, by tissue damage, rapidly activates P2X receptors, which rapidly desensitize [27]. G protein signaling is generally more delayed and prolonged and modulates physiological properties of the neurons. As an example, onset of UTP (P2Y2)-evoked action potentials in vitro took tens of seconds and was far more persistent than P2X-evoked firing [4]. A similar time course was seen for ADP-evoked (P2Y1) Ca++ transients, which in vivo requires hydrolysis of the ATP signal before activation ([28]; this study). Thus, ATP signaling is likely to be more acute, whereas ADP signaling is likely to be more delayed and prolonged.

Our results indicate that P2Y_Gi _receptors negatively regulate the sensitivity of peripheral nociceptive sensory neurons. Given these findings, we were surprised to find that the widely-prescribed anti-thrombotic drug clopidogrel (Plavix), a P2Y12 antagonist, is used extensively in humans with extremely limited reports of pain-related contraindications. It is possible that extensive co-expression of P2Y12 with P2Y13 in sensory neurons, both of which are G_i/o_-coupled ADP receptors, prevents pro-nociceptive consequences of peripheral P2Y12 antagonism in experimental models and in patients using clopidogrel. Furthermore, there is evidence that P2Y12 is required for the expression of neuropathic pain due to a key role in the activation of spinal microglia after nerve injury [29,30]. Because of the powerful actions of spinal microglia in neuropathic pain models, systemic blockade of P2Y12 appears to be protective against persistent pain at the spinal level despite the anti-nociceptive actions of this receptor in primary afferents. Further studies will be required to determine whether P2Y12 antagonism is deleterious in models of persistent pain that lack a significant contribution from microglia.

Opioid receptors are the most extensively studied Gi-coupled receptors in sensory neurons due to their potent anti-nociceptive actions. Interestingly, the pattern of regulation of the P2Y_Gi _receptor mRNAs in response to CFA was grossly similar to regulation of mRNA for the mu opioid receptor [31]. Opiates are generally effective in treating inflammatory pain, however they have potentially dangerous side effects, including tolerance and suppression of respiration and gastrointestinal motility. Several studies indicate that in the peripheral nervous system, opioid receptors are preferentially expressed in peptidergic nociceptors, identified by expression of the neuropeptide CGRP. Wu and colleagues found that both opioid receptor immunoreactivity and opioid inhibition of VDCCs were significantly greater in IB4-negative (presumptive peptidergic) neurons than in IB4-positive neurons, indicating preferential expression of opioid receptors in the peptidergic nociceptors [32]. We found immunoreactivity for P2Y12, P2Y13 and P2Y14 in virtually all small neurons. Inhibitory signaling through P2Y_Gi _receptors is thus likely to impact nociceptive signaling in both peptidergic and non-peptidergic nociceptors. Therefore, it may be possible to improve the treatment of persistent pain by targeting P2Y_Gi _receptors, either alone or in combination with opiates; such combination therapy may allow the use of lower doses (and reduced side-effects) than would be possible when targeting a single receptor.

## Conclusions

We describe functional effects of Gi-coupled P2Y receptors in sensory neurons and implicate these receptors in the regulation of nociceptive signaling. P2Y_Gi _receptors are widely expressed in sensory neurons and inhibit nociceptive signaling. We also found that inflammatory hyperalgesia is reduced in the absence of functional P2Y1. We propose that pro- and anti-nociceptive nucleotide receptors are broadly co-expressed by nociceptive sensory neurons, and that the integration of these opposing signals adjusts nociceptor sensitivity. These signaling pathways are transcriptionally regulated in response to inflammatory injury.

## Methods

### Mice

Adult male wild type C57/Bl6 mice or P2Y1 null mutant mice bred onto the C57Bl6 background were used for all experiments. P2Y1 knockout mice (generously provided by Dr. Beverly Koller, University of North Carolina, Chapel Hill) were maintained as homozygotes and bred normally [33]. All mice were housed in group cages, maintained on a 12:12 hour light-dark cycle in a temperature controlled environment (20.5°C) and given food and water *ad libitum*. These studies were carried out in accordance with the guidelines of the Institutional Animal Care and Use Committee at the University of Pittsburgh and the NIH Guide for the Care and Use of Laboratory Animals.

### Real-Time PCR

Real-time PCR analysis was carried out as previously described [31]. Briefly, L3, L4 and L5 dorsal root ganglia were dissected bilaterally and collected on dry ice. To isolate RNA, frozen tissue samples were placed in 1 ml Trizol reagent (Invitrogen, Carlsbad, CA), homogenized, extracted in chloroform and separated in phase lock gel tubes (Eppendorf, Hamburg, Germany). RNA was precipitated in isopropanol at -20°C for 1 hour then on dry ice for 1 hour, then washed with 75% ethanol and resuspended in water. RNA quality was determined using an Agilent (Palo Alto, CA) 2100 Bioanalyzer according to the manufacturer's instructions and quantity was determined using the 260 nm absorbance recorded by a spectrophotometer. Extracted RNA was treated with DNase (Invitrogen) to remove genomic DNA (1 μl DNase, 2 μl 10× DNase buffer, 0.25 μl RNasin/5 μg RNA in H2O, 20 μl total/reaction). RNA was then reverse-transcribed using Invitrogen Superscript II reverse transcriptase according to the manufacturer's instructions. Negative control reactions were run without RNA to test for contamination. PCR primers were generated using Primer Express software (Applied Biosystems, Foster City, CA) with parameters optimized by the manufacturer. SYBR Green PCR amplification was performed using an Applied Biosystems 5700 real-time thermal cycler controlled by a Dell Latitude laptop computer running ABI Prism 7000 SDS software. After amplification, a dissociation curve was plotted against melting temperature to ensure amplification of a single product. All samples were run in triplicate, and reactions were run without template and with the reverse-transcriptase negative control reaction products as negative controls with every amplification run. Threshold cycle (Ct) values, the cycle number in which SYBR Green fluorescence rises above background, are recorded as a measure of initial template concentration. Relative fold changes in RNA levels were calculated by the ΔΔCt method using p53-glyceraldehyde-3-phosphate dehydrogenase (GAPDH) as a reference standard: Ct values from samples run in triplicate (n = 5 mice/time point, not pooled) were averaged and subtracted from the reference standard, yielding ΔCt. The difference between the ΔCt of the experimental and control groups was then calculated (ΔΔCt). The relative fold change was determined as 2^-ΔΔCt^. Statistical significance was determined by ANOVA using the Statview software package. Data were plotted as the percent change in mRNA levels compared to baseline.

### Inflammation

An emulsion of CFA (heat-killed and dried Mycobacterium tuberculosis in paraffin oil and mannide monooleate; Sigma, St. Louis, MO) was prepared by thoroughly mixing equal volumes of sterile saline and CFA. Mice received a sub-cutaneous injection of the CFA emulsion (20 μl) in the plantar surface of both hindpaws [34]. Animals were deeply anesthetized with Avertin in saline and killed by transcardial perfusion with 4°C isotonic saline at 1, 4 or 15 days after CFA injection.

### Behavioral Analysis

Mice (n = 10/genotype) were placed in individual plexiglass chambers on a glass plate maintained at 30°C and allowed to acclimate for one hour. In all behavioral experiments, the experimenters were blinded to the genotype of the mice and any drug treatment. Paw withdrawal latencies to noxious heat stimulation were measured by applying a radiant heat stimulus (15% intensity on the Hargreaves apparatus; IITC Inc.) to each hindpaw. The heat source was activated with an electric trigger coupled to a timer, and the latency to stimulus response (flinching or lifting the paw) was recorded to the nearest 0.1 second. Nucleotides and P2Y antagonists were purchased from Sigma (IDP) or Tocris.

### Immunohistochemistry

Mice were given an overdose of Avertin anesthetic and killed by transcardial perfusion with 4°C saline followed by 4% paraformaldehyde. DRGs were rapidly dissected, placed in 25% sucrose overnight and frozen in OCT mounting medium. Cell counts were performed in lumbar L4 DRG. Sections were cut at 12 microns on a cryostat and collected on Superfrost microscope slides, then kept at -20°C until used. Slides were placed in blocking solution containing 2% normal donkey serum, 0.2% Triton X-100 in PBS for 30 minutes, then incubated in primary antibody solution overnight at room temperature. Next, slides were washed 3 times for 3 minutes in PBS and incubated for 30 minutes in secondary antibodies diluted 1:500 in blocking solution. Secondary antibodies were donkey anti-rabbit or donkey anti-mouse conjugated to CY3 or CY2 (Jackson Immunoresearch, West Grove PA). Slides were washed 3 times in PBS, dipped in water and coverslipped in glycerol-based fluorescent mounting medium (Dako), then photographed under epifluorescence with a digital camera. Primary antibodies used were rabbit polyclonal anti-P2Y1 (Alomone), anti-P2Y12 (Alomone; 1:200), anti-P2Y13 (Novus; 1:300) and anti-P2Y14 (Affinity Bioreagents; 1:2000). To test for selectivity, sections were processed with antibodies adsorbed against the antigenic peptide, and with secondary antibody alone. No staining was seen in the absence of primary antibody, and preadsorbtion with antigenic peptide abolished staining. Antigenic peptide was not available for P2Y14.

### Calcium Imaging

Isolated neurons were prepared as described [35]. Adult mice were given an overdose of Avertin anesthetic and perfused transcardially with 4°C Ca^++^/Mg^++^-free Hank's basic salt solution (HBSS). All cervical, thoracic and lumbar DRGs were rapidly dissected and cleaned in HBSS. Ca^++ ^imaging was performed 18-24 hours after plating essentially as previously described [36]. Cells were loaded with 2 mM fura-2-AM in HBSS with 5 mg/ml bovine serum albumin for 30 minutes at 37°C, then mounted on a microscope stage with constantly flowing HBSS at 5 ml/minute. Perfusion rate was controlled with a gravity flow system and perfusate temperature was maintained at 30°C using heated stage and in-line heating system (Warner Instruments). Drugs were delivered with a computer-controlled rapid-switching local perfusion system. Firmly-attached, refractile cells were identified as regions of interest in the software (Simple PCI, C-Imaging). Absorbance data at 340 and 380 nm were collected once per second and the relative fluorescence (ratio 340/380) was plotted against time. Calcium transients were examined in response to application of agonists as noted in the figure legends. Calcium responses were quantified as the percentage of cells responding to a given agonist and the amplitude, area, latency and duration of the response using Excel macros written for this purpose.

To evaluate the inhibition of evoked transients by P2Y12, P2Y13 and P2Y14, a 5 second application of 50 mM K^+ ^in HBSS was applied twice with a 5 minute interval to verify reproducible calcium transients, then the appropriate agonist was perfused for 3 minutes before and during the 3^rd ^depolarization. Successive applications of 50 mM K^+ ^were delivered at 5 minute intervals to determine the duration of inhibition. In some experiments, 1 μM capsaicin was applied to determine inhibition in capsaicin-responsive nociceptors. Capsaicin was dissolved in 1-methyl-2-pyrrolidinone as a 10 mM stock solution; 1 μM capsaicin was made fresh daily in HBSS. Pertussis toxin (250 nM; Tocris) was applied to cultures overnight.

## Competing interests

The authors declare that they have no competing interests.

## Authors' contributions

DM initiated the study and prepared the manuscript. Both authors contributed to experimental design and execution, data analysis and editing of the manuscript. Both authors read and approved the final manuscript.
